# Student Reflections on Prosthodontics Patient Encounters: Reversible and Irreversible Procedures: Following Diagnostic Interactions

**DOI:** 10.1002/jdd.13956

**Published:** 2025-06-13

**Authors:** David C. Johnsen, Mai El Najjar, Sally Roushdy, Franciele Floriani

**Affiliations:** ^1^ Department of Pediatric Dentistry Dental Science Building University of Iowa Iowa City Iowa USA; ^2^ Department of Prosthodontics, Dental Science Building University of Iowa Iowa City Iowa USA

**Keywords:** conceptualize, critical thinking, patient interactions

## Abstract

**Purpose:**

Conceptualizing the next patient interaction is done intuitively by the master practitioner for every patient encounter. The project analyzes student reflections following interactions with patients involving reversible and irreversible procedures and follows a project analyzing reflections with patients involving diagnoses. The main purpose was for the student to ask key questions of every patient for the rest of their career. A secondary purpose was to analyze patterns of responses.

**Methods:**

Forty students in Spring 2024 completed the Prosthodontics exercise mostly with reversible or irreversible procedures with the remainder being for examinations. Four open‐ended questions were 1) differentiation from the ideal, 2) desired outcome(s), 3) self‐capabilities, and 4) consequences/prognosis.

**Results:**

Note that, 100% of students responded to all four questions, and over 90% of responses were judged by faculty to be relevant to and appropriate for the theme of each respective question. The authors categorized responses into patterns for each question. The exercise elicited different kinds of thought experiences for students reporting on examinations, reversible procedures, and irreversible procedures. For Questions #1, 2, and 4, for reversible procedures, the focus was more on patient tolerance/acceptance of the appliance. For irreversible procedures, the focus was more on the teeth. Responses were first categorized qualitatively. The difference was statistically significant for Questions #1 and 2. For Question #3, no notable difference was found between reversible and irreversible procedures.

**Conclusions:**

The main purpose was met by engaging students in reflective responses to the four questions. With little literature on the subject, responses to these questions, tell us how students are learning. The collective intuition of students can be converted into a learning guide (learning guidance). The project follows an emulation model for critical thinking.

## 1 Introduction

Reflecting on and conceptualizing the next patient interactions have been shown to be powerful ways of learning [1, 2, 3, 4, 5, 6]. Concepts applicable to reflecting on life are also applicable to reflecting on patient interactions. David Hume said that reflection is at the heart of scholarship [7, 8]. Conceptualizing the next patient interaction is done intuitively by the master practitioner for every patient encounter. Four questions are asked (formally or intuitively) at every patient encounter: 1) How and how much does this situation differ from the ideal? 2) What is the outcome I am seeking at the end of today's encounter? 3) What are my capabilities to successfully manage this situation? 4) What are the consequences/prognosis of this encounter [1, 2]? Preliminary work on student reflections involved each of the four questions just stated with patient interactions involving examinations/diagnoses. In that project using the same four questions as the present project for reversible and irreversible procedures, the format was found to be workable and viewed by students as important for practice [1, 2]. The main purpose of the project was for the student to get into the habit of asking these questions of every patient at every encounter for the rest of their career. A secondary purpose was to analyze patterns of responses. Such an analysis can give insights into how students are thinking during these reflections with the potential for adjusting teaching materials accordingly. Students in the second year of dental school (2/D2) have completed four years of undergraduate studies and one year of dental school. For students conducting examinations for Prosthodontics, the sequence of questions led to responses sequentially reflecting empathy, compassion, reflection, and social projection. An exercise was started in the fall of R2 Year 3/D3 (following simulation in the spring of Year 2/D2) [1] and continued for this part of the project into the spring of Year 3/D3 year. 100% of students completed the exercise citing favorable relevance to practice. All students in the Fall reflected on patients presenting for screening and exams. A JDE paper reported results [1]. In summary of the referenced previous work, the overall goal was to engage students in reflection on patient interactions, a skill the dentist will use for the rest of their careers. Questions were piloted for the level of engagement with the patient and succinctness. Open‐ended questions were selected. The four open‐ended questions were 1) differentiation from the ideal, 2) desired outcome(s), 3) self‐capabilities, and 4) consequences/prognosis. All students were engaged in Prosthodontics diagnosis. 100% of students responded to all four questions. The authors categorized responses into natural patterns for each question. For example, responses to the question on differentiation from the ideal had a strong pattern of empathy for the patient. Responses on desired outcomes had a strong pattern of compassion—empathy with the intent of action. Responses on self‐capabilities had a strong pattern of self‐reflection. Authors separately assigned responses to patterns. The sequence of questions led students to thought experiences from empathy in Question #1, to compassion in Question #2, and to self‐reflection in Question #3 to social projection in Question #4. The exercise was continued in the Spring of Year 3/D3 with a different group of students. For the Spring Year 3/D3 students, five were involved with patients for screening/exam, twenty were for reversible procedures, and fifteen were for irreversible procedures. The purposes of the present project were 1) to reinforce the systematic conceptualization of the next patient encounter with extension from patients receiving examinations to patients receiving reversible (complete dentures, removable partial dentures) and irreversible procedures (crowns, fixed partial dentures); 2) to analyze patterns of responses for examination, reversible procedures, and irreversible procedures. Results can lead to more targeted teaching strategies. With little research on the topic, the collective intuition of students can be converted into a learning guide (learning guidance). The previous work was conducted early in the clinical experience in Prosthodontics where students were conducting examinations. To continue this work, students were asked to reflect on the same four questions with reversible and irreversible procedures.

In addition to previous pilot research, the current study builds on a more general reflection previously reported [1, 2, 3]. A skillset for general reflections is potentially powerful because it can apply to situations beyond dentistry. The skillset itself was established with concepts from outside the health sciences [7, 8, 9, 10]. Written responses gave the faculty member insight into the student's background, values, biases, and mechanisms of introspection [1, 2]. The sequence of student experiences was first an exercise in personal reflection which did not involve patient interactions or simulations [2, 5]. Next and separately, using concepts from the Reflections exercise, the conceptualization of patient interactions was initiated in concept and then with a pilot using forerunners of the four questions used in the present project [1, 2].

While the previous work on conceptualization offered general themes on how students reflect on the next patient visit, the level of understanding about student thought is still considered to be in the preliminary stages. With the present continued work, the intent is to explore additional levels of student thought on patient interactions. The potential is that by unveiling how students think, we can adjust teaching strategies accordingly.

The theme of conceptualization fits into a larger picture of learning guidance and performance assessment in critical thinking [11, 12, 13, 14, 15]. The four questions on conceptualization follow an emulation learning model where the thought process of the expert is derived succinctly enough for the novice to apply to the next patient. In this case, the four questions were derived from highly experienced teacher‐clinicians and summarized into four questions.

## 2 Methods

This observational study was conducted at the University of Iowa, and IRB Exempt Status was granted (#202306291). Results of 43 student exercises were reported in JDE using the same basic methodology as this continuation of the project [1]. The endpoint is for the student to reflect on each of the four questions conceptualizing the next patient interaction: 1) How and how much does this situation differ from the ideal? 2) What is the outcome I am seeking at the end of today's encounter? 3) What are my capabilities to successfully manage this situation? 4) What are the consequences/prognosis of this encounter [1, 2]? Embedding these questions into a habit is seen as emulating the thinking of the master clinician with every patient encounter. To orient students to the exercise, an in‐person session was provided to the students, with examples of written documents explaining the exercise from the pilot project. The current research questions were the same as the four used the previous semester where all the students were conducting examinations. As with the previous project with examinations, students responded to the four questions before meeting with faculty to prepare for the patient. Seven different faculty from the Prosthodontics Department engaged with students to complete respective exercises with responses to the four questions. Data analysis was provided by one faculty from the Prosthodontics Department with regular clinical teaching assignments and familiarity with students and one faculty with experience in designing critical thinking skillsets and assessments. Student/Faculty interactions were similar to interactions in the previous semester except that the procedures were notably different from the previous semester where the students applied questions to patients receiving an examination; the present continuation of the project had mostly patients receiving reversible and irreversible procedures [1]. The difference is that students conducting examinations are looking not only at the dental/oral structures for the replacement of teeth but at the patient's capability to manage the dental procedures as well as the patient's capacity to manage ongoing care. Students conducting reflections on patients receiving reversible or irreversible procedures were looking beyond the management of care (keeping appointments, finances, transportation, etc.), already resolved in the diagnostic exercise, to the patient's ability to accommodate extensive procedures. We did not know how the students would reflect on the anticipation of care delivery—the purpose of the project.

Students entered responses with handwriting on paper for the four questions. While we were not sure of the exact responses, the anticipation was that patterns of responses would emerge. Thus, no categorization of responses was done until after responses were received. The authors met to determine natural patterns as listed in the Tables. For example, for Question #1, “How and how much does this situation differ from the ideal?”, patterns were judged to be “Appliance acceptance/tolerance” and “Focus on the tooth or teeth”. While the primary goal of the project was to engage the student in reflection, and while written responses have limitations, responses were first categorized qualitatively and then subjected to statistical analysis to gain insight into collective student reflections for each question and to gain insights on improving the exercise.

The assessment follows an emulation model previously reported [6, 11, 16] with two stages in the assessment: 1) Did the student “apply” the step in the critical thinking process? 2) Did the student “grasp” the concept in the step in the thought process (in this case, the question) as applied to the patient? The part on “apply” is considered objective and the part on “grasp” is considered subjective. Faculty then make an overall judgment of mastery. Since this is an interactive process, “teaching moments” are available in real‐time should the faculty judge that the student is making good progress but needs additional coaching on a specific point.

## 3 Results

The exercise was completed by 40 students with seven different faculty. All questions were answered by all students. Hand‐written responses were recorded by one faculty member. Uncertainty of a word was rarely noted and there were no instances where the gist of the response was in question. Complete student entries from the first semester were available for comparison with entries from the second semester [1].

The overall result was for students to reflect on questions with little or no overlap in responses among questions. For student responses, results are presented in a qualitative format with statistics used selectively for corroboration. For **Question #1**: How and how much does this situation differ from the ideal?: For reversible procedures, the focus was more on patient tolerance/acceptance of the appliance. For irreversible procedures, the focus was more on the teeth. Examples appear in Table 1.

**TABLE 1:** Question #1: How and how much does this situation differ from the ideal? Sample entries for student responses.

Reversible procedures	Irreversible procedures
This patient has high maxillary + mandibular frenum attachments, which is harder for denture seal.	Slightly overprepped lingual surface
Large gag reflex, a canted occlusal plane, needs a pillow for her back. Exacting personality.	Tipped #31 + 29 make a path of draw challenging.
Pt lives far away and has to drive for 2 h. dependent on the daughter. Lower alveolar ridge not ideal for retention	Flat anatomy from attrition for tooth modification. Esthetics concerns w/frequent pt feedback.
This patient's situation differs greatly from the ideal. She is unable to bite into a consistent position repeatedly—which is very important when adjusting the occlusal guard.	Differs a little bit from ideal—pt had a crown that needed an occlusal rest seat.
More repetition on the mandibular ridge. History of the very same denture for years.	Prept interim >> The tooth I was prepping was rotated.
Over half of the student entries were directed to either appliance acceptance or tolerance or toward a tooth or teeth. The sampling was subjected to the chi‐square test.	

Focus	Reversible	Irreversible
Appliance acceptance/Tolerance	12	1
Tooth/Teeth	3	8

The chi‐square statistic is 10.8. The *p*‐value is 0.001. the result is significant at *p* < 0.05.

For **Question #2**: What is the outcome I am seeking at the end of today's encounter?: More focus was on patient acceptance of reversible procedures compared to irreversible procedures. More focus was on the teeth with irreversible procedures compared to reversible procedures. More focus was on the technical aspects of impressions for reversible procedures than for irreversible procedures. Examples appear in Table 2.

**TABLE 2:** **Question #2**: What is the outcome I am seeking at the end of today's encounter? Sample entries for reversible and irreversible procedures.

Reversible procedures	Irreversible procedures
Pt. was very satisfied with the wax try in & happy. He would be happy getting new dentures, but I'm not sure that pt. understood that dentures take time to adaptation.	Improve pt's understanding in OH & Tx by building a trusting relationship. My goal is to also est an ideal crown prep & temporize to restore esthetics, and function & protect the tooth of focus.
Increase pt comfort in his denture. Encourage pt to continue adjusting to this denture.	To have the patient be satisfied with a gold crown, occlusion, and fit. Cement properly
Delivery of functional dentures that don't irritate the patient and meet esthetic expectations	A well‐fitting provisional crown w/o open margins + no excessive contacts
Retentive max denture + stability of lower denture	Adequate prep + interim that restores function
Dentures fit well, stable, balanced occlusion.	Prep of #29 & provisional. Reviewed oral hygiene habits 7 corrected
Over half of the student entries were directed to either appliance acceptance/tolerance/technical aspect of the impression or toward a tooth/teeth. The sampling was subjected to the chi‐square test.	

Focus	Reversible	Irreversible
appliance acceptance/tolerance/technical	16	1
Tooth/Teeth	1	11

The chi‐square statistic is 19.0271. The *p*‐value is .000013. Significant at *p* < .05.

For **Question #3**: What are my capabilities to successfully manage this situation?: Regardless of whether the procedure was reversible or irreversible, there were fewer differences between reversible and irreversible procedures for this question. The focus was on the student's introspection on themselves rather than focus on the patient as with Questions 1,2 and 4. Examples appear in Table 3.

**TABLE 3:** For Question #3, What are my capabilities to successfully manage this situation? Sample entries for reversible and irreversible procedures.

Reversible procedures	Irreversible procedures
Staying calm and patient and maintaining proper communication with the patient and faculty.	I feel capable despite it being my first delivery.
Good—this is the second patient I will be performing this procedure on.	Hand skills + knowledge from simulation
Lowering pt expectations and explaining limitations of CUCL—express denture adhesive with help.	Establish rapport early on, seek understanding, listen/empathize & motivate pt.
I have the ability to read people and adjust according to how they present at the appointment.	I feel that I am capable of providing good clinical treatment as long as I set the pt expectations appropriately.
To see areas of improvement and learning my limitations	Some tips and helpful instruction from the faculties

For **Question #4**: What are the consequences/prognosis of this encounter?: More focus was on patient acceptance of reversible procedures compared to irreversible procedures. More focus was on the teeth with irreversible procedures compared to reversible procedures. Examples appear in Table 4.

**TABLE 4:** **Question #4**: What are the consequences/prognosis of this encounter? Sample entries for reversible and irreversible procedures.

Reversible procedures	Irreversible procedures
If pt. leaves without adjustment it could level to some spots	#21 RCT w/little remaining tooth structure, requires a crown to avoid fracture
Pt is committed to adapting to the prosthesis.	Good with nice preparation. Need to set good margins for the lab to fabricate [porcelain fused to metal, gold or all ceramic] crown.
Pt being frustrated that CUCL will always gag, pt understands I'm doing my best to ensure comfort, esthetics, occlusion, and phonetics.	Prognosis is good. Provisional is temporarily cemented well adherent to our recommendations of practices & I see a good prognosis for her future RPD since she has good oral hygiene.
May need to reline denture to retention	Consequences include potential wear on opposing teeth (Zr Crown) & cost. The prognosis is expected to be favorable, providing improved aesthetics & function.
Potentially poor speaking ability with denture	Fair prognosis of tooth #8 with improved OH, routine dental exams, compliance & est dental home

Assessment for critical thinking is inherently a work in progress with little literature as a guide. Assessment has an objective component and a subjective component [11]. The objective component is, “Did the student apply each step in the thought process?”—in this exercise, did the student address each of the four questions? The subjective component is, “Does the student's response relate to the question and the patient situation?” The faculty then made a judgment. With few “right” answers, students are given latitude in responses. The larger issue is the systematic asking of each question with each patient. For faculty assessments, all students applied the step for each question. Seven different faculty conducted assessments. Table 5 For the student assessments, thirty‐seven of forty students were judged by faculty to “Apply” the question to the patient. The remaining three assessments were completely blank. Thirty‐four of thirty‐seven faculty assessed overall Mastery with all making entries judging as “Yes” for mastery. While progress has been made on the assessment, the assessment for this exercise and on critical thinking areas in general is an area for further work. In one sense, each question leads to a descriptor in that the question identifies an area of focus.

**TABLE 5:** Number of students assessed by faculty.

Assessed “Applied”	37
Assessed “Grasped”	33
Assessed “Mastery” as “Yes”	34
Assessment of “Mastery” Blank	3
Assessment completely blank	3

## 4 Discussion

Reflection is a powerful tool to engage the patient in the dentist‐patient interaction and is reinforced with this project. We are not aware of a previous project for students systematically engaging in reflections at the point of patient interaction on the four topics selected. Conceptualization is a complex set of mental activities leading from more abstract to more tangible thought experiences; key thought experiences elicited from conceptualization can be empathy, compassion, focused reflection, and social projection [1, 12]. Reflection is essential as the vast array of variables unfolds on a moment‐by‐moment basis. The agility of the practitioner's mind as patient encounters unfold will be a factor in success in patient care. Singer: “Students have attitudes, beliefs, and expectations about learning that can influence their behavior and performance in courses. In addition, differences in metacognitive ability translate into differences in students’ learning outcomes. Students who are more metacognitive are better students overall” [9]. Reflection can shape attitudes and instill habits for practice.

Interactions were considered by faculty as positive with a 100% response rate. The 100% response rate previously found with exams and with a 100% response rate for irreversible and reversible procedures is further interpreted as reinforcement to continue the evolution of the format. The succinct, but probing responses by students are also interpreted as an indication of a high focus on the person/patient beyond simulation.

While no two individual responses were the same for the 160 total responses in this part of the project, responses did fall into categories for each question. The array of responses falling into general categories is seen as an opportunity for customized teaching moments. If all were the same, such an opportunity might be standardized. One interpretation is that students reflected individually on the general theme of the question and within the boundaries of the topic. Results are viewed as a largely qualitative review of responses. While faculty agreed on differences between the kinds of responses for reversible and irreversible procedures, the differences are not seen as extreme. Thus, analysis is seen as mostly qualitative with statistics seen as corroborative.

Since the definition of critical thinking considered in this project is “The art of analyzing one's thought process with the intent of improving it.”, the question arises on the extent to which “art” can be quantified [13]. The concept of drawing on extensive observations as a basis for describing a scientific phenomenon goes back to Darwin and beyond [17]. While some quantification is offered, caution is suggested in taking conclusions beyond the power of the method. A central concept reinforced in the exercise is the seeking of alternatives from which the dentist and patient agree on a plan; alternatives have inherent limitations for quantification. The emphasis of this project is to offer an extensive set of categorized observations to describe a learning concept and then support it with statistics as in the Tables. The central concept is that the thought process is the learning outcome, the learning guide, and the assessment instrument. This concept is recent in the dental education literature with an invitation for further work. We were not aware of the literature before the present sequence of studies offering some quantification of a thought process.

With caution due to the modest sample size, there are, nonetheless, trends worth noting in this area with little literature. In an area with little literature, the study is seen as a start, balanced with caution of long‐term conclusions with a limited sample size. Of potential importance in designing educational strategies is the train of thought from the initial exam to the divergent thinking with reversible and irreversible procedures. At the examination, the student seeks to note barriers to care—finances, transportation, potential compliance, etc. Once the student judges that the patient will be accommodating for care, the focus shifts to the oral conditions. For reversible procedures, the focus is more often on the acceptance/tolerance of the appliance and on the technical aspects of the impression. For irreversible procedures, the focus is more often on aspects of the teeth for preparation as well as fixed appliance fit and adaptation. While the differences in student thinking between patients with reversible and irreversible procedures are not extreme, the anticipation of this train of thinking allows instructional material for the student to anticipate the train of thought rather than wait for the student to discover the concept (or not) intuitively. With little literature on the subject, one interpretation is that students are telling us how they are learning. The collective intuition of students can be converted into a learning guide or general learning guidance. Caution also applies to the statistical tests; while the statistical significance is noteworthy, the numbers are small. As with qualitative analysis, trends are promising for the exploration of teaching methodology. An agenda item for future projects is progress regarding faculty consistency to guide learning and assess performance with the array of student responses. We are reluctant to radically change the format with the individualized teaching moments. The project follows an emulation model for critical thinking [6, 11].

Historically, Prosthodontics was divided into Fixed prosthodontics and Removable Prosthodontics. Student comments reflect the different thinking for dealing with patients receiving fixed and removable appliances. While the responses have some specific technical terms about prosthodontics, the concept of generating divergent responses for the same question extends to other disciplines. For example, in risk assessment for caries or periodontitis, the divergence comes with the disease status. With disease progression for either caries or periodontitis, the line of thinking will be about reigning in the disease more than about conducting procedures with an escalation of interventions. With minimum disease progression, the thinking will assume that the disease of caries or periodontitis is being managed, and thinking will be more about procedures for improving the oral condition of the patient, for example, esthetics, crown lengthening, implants, etc. [18]. Another example is the question asked of medically compromised patients, “Which data are more important and why?” For patients with extensive health or social conditions, the responses are more about mitigating barriers to care than about dental procedures and more about the person's health or social barriers and less about the teeth; for patients with minimum barriers to care either medically or socially, responses are more about the teeth [16]. The present exercise is seen as having value for the specific questions to become ubiquitous for the student and as having value for considering alternatives as well as “answers”.

In this project, educational concepts to reconcile within the general realm of critical thinking are learning outcomes, assessment, critical thinking as an art, and novice to expert; the combination is for a separate essay [11]. From novice to expert, the novice needs some structure while structure may inhibit the expert [10]. Prototypes are sparse for faculty assessment for explicit exercises in critical thinking. The use of “Apply” for the objective component and the use of “Grasp” for the subjective component are works in progress [14, 15, 16]. Systematically tracking the emulation of the designated thought process by the student is central to an evolving model. Implementation of the “Apply”/“Grasp” approach by over 90% of faculty is seen as progress in this evolution. Consistency between assessment of each step and mastery is also seen as progress in this evolution. For this exercise, the lack of “right” answers would seem to reflect the practice situation where “right” answers can be elusive.

The practical benefit of the exercise is the use of the four questions in practice. The questions are derived from master teachers and clinicians for use with every patient for the rest of their respective careers. With little or no literature on the subject, the authors see this as an initial step to invite further corroboration in an area where we are improving critical thinking as an “art” with the invitation for more widespread corroboration with quantification as supportive data where possible.

## 5 Conclusions

This observational study elicited a wide reflection on four topics encountered by the general dentist at each patient interaction: discerning how and how much the situation differs from ideal; visualizing the outcome of the patient encounter; self‐assessing one's capabilities; and projecting prognosis and consequences. With the modest sample size, caution is taken in drawing wide conclusions. Categories of responses with little or no overlap among questions are interpreted as offering a range of perspectives on patient care. The format was succinct with acceptance by students and faculty. The skillset provides an educational tool to guide learning and assess student introspection and critical thinking. All students provided critical evaluation and reflection with four key questions to begin a prosthodontics treatment. For patients with reversible procedures, students more often looked for accommodation or tolerance to the appliance. For reversible procedures, students more often focused on perspectives related to the tooth or teeth. Qualitative differences in reflections on patients with examinations, reversible, and irreversible procedures are interpreted to offer opportunities prospectively to guide learning. The students are telling us how they are learning. The emulation model seems suited to reflective learning.

## Conflicts of Interest

The authors declare no conflicts of interest.
